# Comparisons of the Effect of Different Metal Oxide Nanoparticles on the Root and Shoot Growth under Shaking and Non-Shaking Incubation, Different Plants, and Binary Mixture Conditions

**DOI:** 10.3390/nano11071653

**Published:** 2021-06-23

**Authors:** In Chul Kong, Kyung-Seok Ko, Dong-Chan Koh

**Affiliations:** 1 Department of Environmental Engineering, Yeungnam University, Gyungsan 38541, Korea; ickong@ynu.ac.kr; 2 Geologic Environment Division, Korea Institute of Geoscience & Mineral Resources (KIGAM), Daejeon 34132, Korea; chankoh@kigam.re.kr

**Keywords:** binary mixture, incubation condition, nanoparticles, plant type, toxicity

## Abstract

We evaluated the toxicity of five metal oxide nanoparticles (NPs) in single or binary mixtures based on root and shoot growth of two plant species under non-shaking and shaking conditions. The effects of NPs on root and shoot growth differed depending on the NP type, incubation condition, and plant type. The half maximal effective concentration (EC_50_) of NPs based on root growth were significantly lower, by 2.6–9.8 times, under shaking than non-shaking conditions (*p* = 0.0138). The magnitude of the effects of NPs followed the order CuO > ZnO > NiO >> Al_2_O_3_, TiO_2_. In addition, *Lactuca sativa* L. was more sensitive to the tested NPs than *Raphanus sativus* L., with an EC_50_ 0.2–0.7 times lower (*p* = 0.0267). The observed effects of 12 combinations of binary NP mixtures were slightly, albeit non-significantly, lower than expected, indicative of an additive effect of the individual NPs in the mixtures. The results emphasize the importance of careful plant model selection, appropriate application of incubation conditions, and consideration of chemical mixtures rather than single compounds when evaluating the effects of metal oxide NPs.

## 1. Introduction

The application of nanotechnology is continuously growing and its development is expected to have positive impacts on society, particularly in the fields of engineering and technology [1]. However, the rapid development and extensive commercial applications of engineered NPs can lead to their discharge into the environment, especially into soil and water, via various pathways [2,3,4,5,6]. Nano-sized (10^−7^ to 10^−9^ m) particles have a variety of characteristics in terms of their diffusion rate, high-reaction surface area-to-volume ratio, and reactivity in the liquid and gas phases, providing more advanced or novel properties compared to their bulk counterparts [7]. Given these unique characteristics, various types of NPs, especially metal-based NPs, have been manufactured and are commercially applied for use in biomedical and industrial settings. Nano-products are widely applied in many fields, including nanofiber materials, tennis racquets/balls, glass coatings, metal corrosion inhibitors, cosmetics, pigments, antibacterial agents, catalysts, energy storage systems, medical devices, and sensors [8,9,10]. With the production of NPs with diverse characteristics, NP applications will continue to expand in the future.

The high production volume and number of engineered NPs can lead to potential human health and environmental problems following either deliberate or accidental releases of NPs into the environment. These effects are generally caused by their unique physicochemical characteristics [11,12,13,14,15]. Researchers have reported various negative effects of NPs that vary according to their particle size, particle shape, surface coating, and capping [16,17]. For example, many engineered NPs are released into the soil system due to the use of biosolids and fertilizers, and are emitted from sewage treatment plants and animal husbandry facilities [7]. Some of these NPs are inevitably released into rivers, lakes, and groundwater, and affect the quality of drinking water, leading to detrimental effects on aquatic ecosystems or human health [18,19]. Although the mechanisms of these effects are not well understood, with many contradictory findings reported, the commonly proposed mechanisms responsible for the negative effects of NPs are related to the presence of solubilized metal ions and their uptake into cells, followed by disruption of the cell membrane, DNA damage, enzyme deactivation, and oxidation by reactive oxygen species (ROS) [20,21,22]. This oxidative damage can be protected by scavenging free radicals of ROS using antioxidant reactive systems in living organisms [23].

Various organisms, and the metabolic processes of plants, microorganisms, earthworms, and arthropods, have been studied to evaluate the effects of NPs [10,21,24,25,26,27]. Among the various study models, the root and shoot growth of plants respond rapidly to acute NP toxicity; seed germination, biomass, and leaf surface area are also affected by NP exposure [28,29,30,31]. Many studies have demonstrated different or opposing outcomes depending on the test organisms [32,33]. For example, some studies have observed negative effects of TiO_2_ and ZnO NPs among microalgae, crustaceans, and bacteria, while others have shown the opposite outcomes. The effects of NPs may vary depending on the test conditions, even when the same organism or endpoint is adopted. Therefore, it is important to characterize the sensitivity of test organisms under various incubation conditions when examining the effects of NPs. Toxicity studies are often performed using only a single chemical rather than chemical mixtures [34], which would reflect a more realistic situation [35]. Therefore, analyses of mixtures are also needed for accurate toxicity assessments. Because the effects and fate of NPs in the environment may also vary with respect to their physicochemical characteristics, such properties also need to be examined.

To address these gaps in knowledge, we performed biological toxicity experiments under various conditions to aid in evaluations of the impacts of NPs on the soil and water environment. To this end, we compared the effects of five metal oxide NPs (commercially available CuO, NiO, ZnO, TiO_2_, and Al_2_O_3_ NPs), alone and in binary mixtures, based on the root and shoot growth of two plant species under different incubation conditions.

## 2. Materials and Methods

### 2.1. Test Materials, Preparation, and Analysis

Five types of commercially available metal oxide NPs were tested: CuO (30–50 nm), ZnO (40–100 nm), NiO (8–20 nm), TiO_2_ (<25 nm), and Al_2_O_3_ (40–50 nm) (obtained from Nanostructured and Amorphous Materials (Houston, TX, USA) and Alfa Aesar (Tewksbury, MA, USA)). To ensure the uniform dispersion of NPs, a high-concentration solution was prepared and diluted with distilled water for 30 min using ultrasonication (Daihan Scientific Co., Ltd., Wonju, Korea). The NPs were suspended directly in deionized water and dispersed via ultrasonication for 10 min before use.

As the two test species, seeds of *Lactuca sativa* L. and *Raphanus sativus* L., produced commercially (Nongwoobio Co., Suwon, Korea) were purchased from a local seed store. The seeds were sterilized with 3% H_2_O_2_ and washed three times with sterile water to facilitate the germination process. All other chemicals were reagent-grade and were purchased from Sigma-Aldrich (St. Louis, MO, USA).

From the dose–response data, we calculated the half maximal effective concentration (EC_50_) using the trimmed Spearman–Karber method, distributed by the United States Environmental Protection Agency (US EPA) Center for Exposure Assessment Modeling. Statistical significance (95% confidence level) among the experimental groups was calculated using Student’s *t*-test (http://www.graphpad.com accessed on January–April 2021).

### 2.2. Effects of Incubation Conditions on Root and Shoot Growth

Germinated seeds were transferred to serum vials containing 30 mL of test solution and incubated at 25 °C under non-shaking and shaking (70 rpm) conditions to compare the effects of different incubation conditions on root and shoot growth. The test concentration ranges for each metal oxide NP were established based on a preliminary test as follows: non-shaking conditions, CuO 0–5 mg/L, ZnO 0–10 mg/L, NiO 0–20 mg/L, TiO_2_ 1000 mg/L, and Al_2_O_3_ 1000 mg/L; shaking conditions, CuO 0–1 mg/L, ZnO 0–2 mg/L, NiO 0–5 mg/L, TiO_2_, and Al_2_O_3_ 1000 mg/L.

After four days of cultivation, the root and shoot lengths of seedlings were measured from their junctions to the longest tip. The root and shoot measurements for each test condition were expressed as the percentage inhibition (%) of the relative root length (RRL) or relative shoot length (RSL) compared to the control.

Following this evaluation, the differences in root and shoot growth in the two plant species (*L. sativa* and *R. sativus*) were evaluated under shaking, which was considered to be the more suitable incubation condition for assessing the toxicity of the partially soluble NPs. At the end of the incubation period, the solution samples were filtered (0.45 μm) to determine the concentration of dissolved metal ions using an inductively coupled plasma optical emission spectrometer (Optima 7300DV; Perkin-Elmer Inc., Shelton, CT, USA).

### 2.3. Effects of Binary NP Mixtures on Root Growth

The effects of binary mixtures of three NPs (i.e., equal mixtures of two concentrations of each of CuO, ZnO, and NiO NP; TiO_2_ and Al_2_O_3_ NPs were excluded for their lower sensitivity), for a total of 12 mixtures, were tested based on the root growth of *L. sativa* and *R. sativus*. For a comparative evaluation of the binary mixtures, we used a model based on probability theory to calculate the expected inhibition, P(E), of each mixture: P(E) = P*_x_* + P*_y_* − (P*_x_*P*_y_*/100), where P*_x_* and P*_y_* are the inhibition caused by NPs *x* and *y* [36]. Then, we compared the calculated P(E) with the experimentally observed inhibition, P(O). Then, the interaction effects of the mixtures (synergistic, antagonistic, or additive) were determined based on statistical analysis. When P(O) was significantly higher or lower than P(E) (*p* < 0.05; null hypothesis), the effect was considered to be synergistic or antagonistic, respectively. By contrast, no significant difference between P(O) and P(E) (*p* > 0.05) indicated an additive effect. The concentration ranges of the 12 mixtures for each plant were determined based on preliminary test results (Supplementary Table S1).

## 3. Results

### 3.1. Effects of Non-Shaking and Shaking Incubation Conditions on Root and Shoot Growth

**Figure 1:**
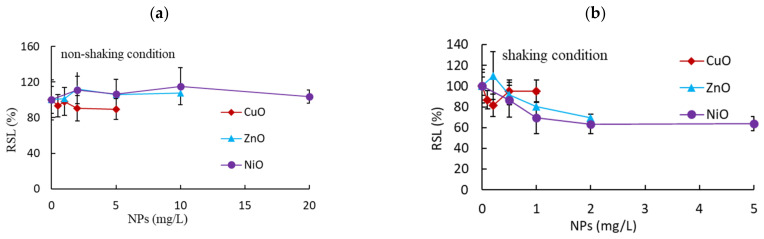
Effects of nanoparticle (NP) exposure on relative shoot growth (RSL) under (**a**) non-shaking and (**b**) shaking conditions.

We first evaluated the influence of incubation conditions (non-shaking and shaking) on the toxic effects of CuO, ZnO, NiO, TiO_2_, and Al_2_O_3_ NPs based on the root and shoot growth of *L. sativa* seedlings. To reduce experimental errors, we only tested seedlings with a root length of 2.0–2.6 cm. After four days of incubation, the root and shoot lengths of the non-exposed control group were 64 ± 6.3 and 14 ± 3.7 mm, respectively. No considerable inhibition of shoot growth was observed under non-shaking conditions, with RSLs of 90 ± 11.5%, 107 ± 11.0%, and 104 ± 7.5% of the control at the maximum exposure concentrations of CuO (5 mg/L), ZnO (10 mg/L), and NiO (20 mg/L), respectively (Figure 1a). Slightly higher inhibition of shoot growth was observed under shaking conditions, with 95 ± 10.9%, 70 ± 12.4%, and 64 ± 6.8% of the RSL achieved at the maximum exposure concentration of CuO (1 mg/L), ZnO (2 mg/L), and NiO (5 mg/L), respectively (Figure 1b). The effects of 2 mg/L NiO and ZnO NPs differed significantly between the shaking and non-shaking conditions (*p* < 0.0290), with an RSL 1.7-fold greater under the shaking than the non-shaking condition. RSL was in the range of 21–28% at the maximum exposure concentrations of CuO (1 mg/L), ZnO (2 mg/L), and NiO (5 mg/L) under shaking incubation conditions (Table 1). By contrast, at the maximum exposure concentrations for TiO_2_ and Al_2_O_3_ (1000 mg/L), the RSLs were 106 ± 11.7% and 108 ± 22.0% under non-shaking conditions and 84 ± 13.0% and 89 ± 12.3% under shaking conditions, respectively (Table 2).

**Figure 2:**
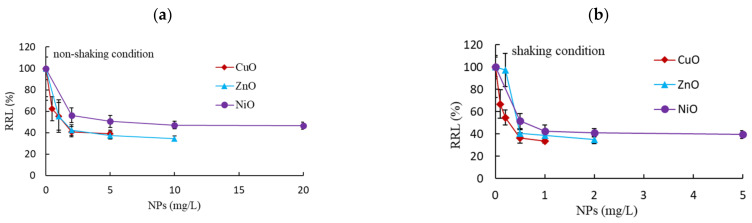
Effects of nanoparticle (NP) exposure on relative root growth (RRL) under (**a**) non-shaking and (**b**) shaking conditions.

Unlike the RSL, RRL showed significant differences between the shaking and non-shaking conditions (Figure 2). For example, at the maximum exposure concentrations, the RRLs under non-shaking conditions were 55 ± 12.9%, 42 ± 5.0%, and 51 ± 5.1% for CuO (5 mg/L), ZnO (10 mg/L), and NiO (20 mg/L), respectively, and 34 ± 2.0%, 35 ± 3.4%, and 40 ± 3.4% for CuO (1 mg/L), ZnO (2 mg/L), and NiO (5 mg/L) under shaking conditions, respectively. Root growth was inhibited less at low exposure concentrations, with RRLs in the range of 41–55% at 1 mg/L and 33–56% at 0.5 mg/L for CuO, ZnO, and NiO NPs under non-shaking and shaking conditions, respectively (Figure 2). After rapid initial inhibition, no further apparent inhibition in RRL was observed in the tested concentrations. Moreover, the RRLs after exposure to 1000 mg/L of TiO_2_ and Al_2_O_3_ were 99 ± 18.8% and 86 ± 26.3% under shaking conditions, and 65 ± 16.4% and 56 ± 10.1% under non-shaking conditions, respectively (Table 2).

The EC_50_ values calculated based on root growth followed the order CuO (1.28 mg/L) > ZnO (1.31 mg/L) > NiO (5.57 mg/L) under non-shaking conditions and CuO (0.25 mg/L) > ZnO (0.50 mg/L) > NiO (0.57 mg/L) under shaking conditions. The EC_50_ values were 2.6–9.8-fold higher under non-shaking conditions than under shaking conditions (*p* = 0.0138) (Table 1).

### 3.2. Comparison of the Effects of NPs on Two Plant Species

**Figure 3:**
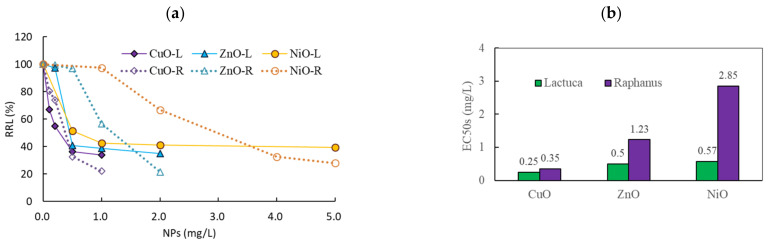
Effects of nanoparticle (NP) exposure on the root growth of *Lactuca sativa* and *Raphanus sativus* under shaking incubation conditions based on (**a**) relative root growth and (**b**) EC_50_. In (**a**), L and R represent *L. sativa* and *R. sativus*, respectively.

We compared the effects of CuO (0–1 mg/L), ZnO (0–2 mg/L), and NiO (0–5 mg/L) NPs on root growth in *L. sativa* and *R. sativus* under shaking conditions. During the incubation period, the root length of *R. sativus* (range: 115–152 mm) was 2-fold longer than that of *L. sativa* (range: 61–74 mm) in the control. When exposed to NPs, the RRLs of *L. sativa* and *R. sativus* were 34–97% and 21–115% those of the control plants, respectively. Strong inhibition of root growth was observed at low exposure concentrations in both species (Figure 3a). At the maximum exposure concentrations of CuO (1 mg/L), ZnO (2 mg/L), and NiO (5 mg/L) NPs, the RRLs of *L. sativa* were 34 ± 2.0%, 35 ± 3.4%, and 40 ± 3.4%, respectively, whereas those of *R. sativus* were 22 ± 3.0%, 21 ± 5.5%, and 28 ± 5.7%, respectively; overall, the RRL of *L. sativa* was approximately 1.5-fold higher than that of *R. sativus*. No considerable negative effects or differences in shoot growth were observed under the tested concentrations, and shoot growth was within 74% of the control under all conditions in both species. The EC_50_ values of CuO, ZnO, and NiO on root growth in *L. sativa* were 0.25 (0.18–0.37), 0.50 (0.36–0.70), and 0.57 (0.32–1.02) mg/L, respectively, while those of *R. sativus* were 0.35 (0.29–0.42), 1.23 (1.10–1.38), and 2.85 (2.48–3.27) mg/L, respectively (Figure 3b); the EC_50_ values in *R. sativus* were 1.4- to 5.0-fold greater than those in *L. sativa* (*p* = 0.0267).

Soluble metal concentrations were measured to evaluate the impact of solubilization from the metal oxide NPs on the root growth of the two plant species (Supplementary Table S2). Low dissolved metal concentrations were detected, within the ranges of 0.8–6.6% (average: 3.8%) and 0.2–5.5% (average: 2.4%) for *L. sativa* and *R. sativus*, respectively.

### 3.3. Effects of Binary NP Mixtures on the Root Growth of L. sativa and R. sativus

**Figure 4:**
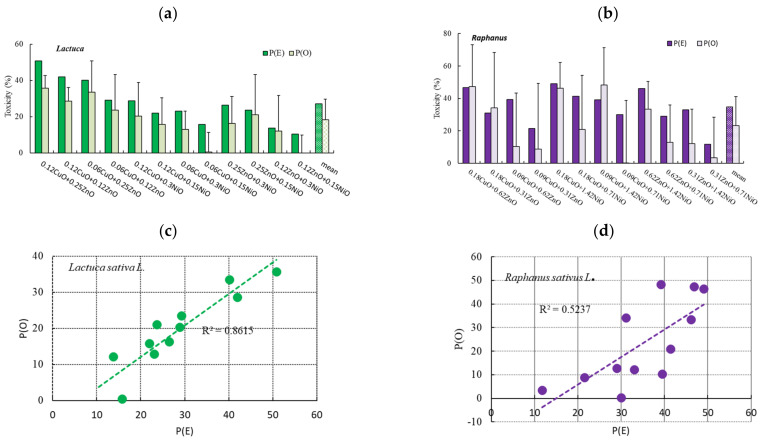
Comparison of the observed (P(O)) and expected (P(E)) inhibitory effects of binary nanoparticle (NP) mixtures on root growth in (**a**) *Lactuca sativa* and (**b**) *Raphanus sativus*. Correlation between P(O) and P(E) for (**c**) *L. sativa* and (**d**) *R. sativus*. In (**a**), the labels show the constituents and concentrations of the binary mixtures, e.g., 0.12 CuO + 0.25 ZnO represents CuO and ZnO at final concentrations of 0.12 and 0.25 mg/L, respectively.

The effects of binary mixtures of three NPs on root growth were investigated using equal mixtures of two concentrations, based on the EC_50_ values of single NP exposure. In *L. sativa*, we tested concentrations of 0.06 and 0.12 mg/L CuO, 0.25 and 0.50 mg/L ZnO, and 0.15 and 0.30 mg/L NiO, and in *R. sativus*, 0.09 and 0.18 mg/L CuO, 0.31 and 0.62 mg/L ZnO, 0.71 and 1.42 mg/L NiO (Figure 4a,b). Due to the non-significant effects of single NPs on shoot growth, the 12 binary NP mixtures were only tested using root growth. The average root lengths of *L. sativa* and *R. sativus* were approximately 66–70 and 81–117 mm in the control group, respectively, but 44–67 mm (64–100% of the control) and 52–117 mm (44–100% of the control) in the exposed groups, respectively. The greatest root growth inhibition was observed at 0.12 mg/L CuO + 0.25 mg/L ZnO (40% inhibition) in *L. sativa*, and 0.18 mg/L CuO + 1.42 mg/L NiO (44% inhibition) in *R. sativus* (Figure 4a,b).

The P(O) and P(E) values of the 12 binary mixtures on root growth were within the range −0.2–35.7% (average: 18 ± 11.4%) and 10.5–50.8% (average: 27 ± 12.1%) in *L. sativa*, and 0.2–48.4% (average: 23 ± 17.9%) and 11.7–49.1% (average: 35 ± 11.1%) in *R. sativus* (Figure 4a,b). The P(O)-to-P(E) ratios were in the ranges of 0.0–0.9 (average: 0.62; *p* = 0.0591–0.9250) for *L. sativa* and 0.0–1.2 (average: 0.61; *p* = 0.2948–0.9786) for *R. sativus*. The correlation coefficients (R^2^) of the relationship between P(O) and P(E) were 0.8615 and 0.5237 for *L. sativa* and *R. sativus*, respectively (Figure 4c,d).

## 4. Discussion

Given the increased use of NPs it is necessary to evaluate their negative effects using suitable test organisms under appropriate conditions. Plants represent simple, reproducible, and cost-effective test organisms, and have been used widely for the bioassessment of environmental contaminants [37]. In particular, *L. sativa* and *R. sativus* are important crops that are relatively sensitive to toxic chemicals, leading to their widespread use in standard toxicity tests [38,39,40]. Various vegetative endpoints in plants can be used to examine the effects of NPs; however, conventional conditions for bioassays, designed to test soluble chemicals in solution, may not be suitable for the assessment of NPs due to their partial solubility. Therefore, we evaluated the effects of shaking and non-shaking conditions for the assessment of partially soluble NPs (CuO, ZnO, NiO, TiO_2_, and Al_2_O_3_; alone or in binary mixtures) based on root and shoot growth in *L. sativa* and *R. sativus*.

Under all test conditions, TiO_2_, and Al_2_O_3_ NPs showed weak inhibitory effects compared to CuO, NiO, and ZnO NPs; therefore, we mainly focused on the effects of CuO, NiO, and ZnO NPs. NPs showed no significant inhibition of shoot growth under non-shaking conditions, and a slight inhibition of shoot growth under shaking conditions. Interestingly, incubation condition (shaking vs. non-shaking) had a significant effect on the influence of NPs on shoot growth (*p* < 0.0290). No positive correlations were observed between root and shoot growth (R^2^ = 0.3182 for shaking, 0.0044 for non-shaking; Supplementary Figure S1), possibly due to the contact between tissue and NPs or the partial solubility of NPs. In particular, the weak correlation under non-shaking conditions may be the result of the almost total lack of inhibition of NPs on shoot growth.

In contrast to the effect on shoot growth, NPs showed a strong inhibition on root growth. Moreover, significant differences between the shaking and non-shaking incubation conditions were observed. Under both conditions, the inhibition based on EC_50_ values followed the order CuO > ZnO > NiO. The EC_50_ values on root growth were about 6-fold higher under the non-shaking condition than under the shaking condition. For all of the test NPs, a considerable significant increase in inhibition was observed under shaking conditions (*p* = 0.0138). This stronger inhibition effect on root growth observed under shaking conditions was likely due to the increase in contact between roots and the partially soluble NPs, morphological effects, and differences in dissolved metal concentrations; such changes could influence the effects and fate of NPs in the environment and their interactions with organisms [41]. Kim et al. [11] reported that the microbial test method (i.e., liquid suspension or in agar) profoundly influenced the effects of Ag NPs. Therefore, methods designed for soluble contaminants need to be modified to properly assess the toxicity of partially soluble NPs. Interestingly, compared to the non-shaking condition, root growth increased by approximately 1.5-fold after exposure to 1000 mg/L TiO_2_ or Al_2_O_3_ under the shaking condition. The difference between toxicity or growth stimulation for plants may be partly related to the transformation of different types of NPs. For example, TiO_2_ NPs are stable and remain in their unaltered form in plants, whereas NPs such as ZnO, CuO, and NiO are able to transform, resulting in differences in the accumulation of different chemical forms and their bioavailability in plants [5,42,43]. Due to the non-significant effects of TiO_2_ and Al_2_O_3_ on shoot growth under both shaking and non-shaking conditions, EC_50_ values could not be calculated from the tested concentrations.

Some researchers have suggested that the toxicity of NPs is mainly due to the solubilized metal ions of NPs [44,45], but others have reported the opposite results [46]. Thus, we measured soluble metal concentrations to evaluate the influence of solubilized metals from metal oxide NPs on root growth (Supplementary Table S2). No consistent patterns were observed and low concentrations of dissolved metals were detected, with a range of 0.8–6.6% (average: 3.8%) for *L. sativa*. In our previous study, the soluble metal concentration of NPs under non-shaking conditions was in the range of 2.4–9.5%; therefore, no considerable differences were observed between the two incubation conditions [47]. As in previous studies [18,48], soluble metals likely contributed minimally to root growth inhibition. Rather, the main cause of the differences in root growth inhibition between the incubation conditions was likely the result of enhanced contact between roots and NPs caused by the shaking.

In our previous research on the effects of metal in eight plant species, *L. sativa*, *R. sativus*, *Cucumis*, *Cardamine*, and *Brassica* were the most sensitive based on EC_50_ values [39]. Based on these results, we selected *L. sativa* and *R. sativus* for the present comparison of the effects of NPs on root growth. Root growth inhibition varied with increasing NP concentration, depending on the NP and plant species. Based on the EC_50_ values, root growth inhibition in both species increased in the order CuO *>* ZnO *>* NiO. Moreover, *L. sativa* (average root growth EC_50_ = 0.44 ± 0.168 mg/L) was more sensitive than *R. sativus* (average root growth EC_50_ = 1.48 ± 1.268 mg/L) to the three tested NPs. These findings suggest different effects on root growth according to NP and plant species. Plant uptake of NPs is affected by multiple factors, such as particle size, morphology, exposure conditions, plant species and growth stage, and root integrity and rhizosphere processes [5]. Furthermore, the influence of plant species on plant root uptake of NPs is complicated due to differences in root exudates, plant physiology, and metabolic function, which further affect the size, surface charge, and speciation of NPs [49,50]. Therefore, the use of only one plant species is likely insufficient for accurate assessments of the effects of NPs. Our findings support the use of combinations of potential toxicants in a variety of plant species to study species-dependent NP uptake.

In this study, the solubilized metal concentrations in the solutions with different plant species were measured to determine the contribution of the soluble metal ions of each NP on plant activity. No consistent patterns were observed and low concentrations of dissolved metals were detected for both *L. sativa* (average: 3.8%) and *R. sativus* (average: 2.4%) (Supplementary Table S2). Overall, slightly less soluble metals were observed with *R. sativus* (e.g., 156 ± 16 µg/L Ni for *L. sativa* vs. 37 ± 20 µg/L Ni for *R. sativus* at 5 mg/L NiO NPs), and there were no apparent differences in soluble metal concentrations in the different plant root growth experiments. Previous investigations in our laboratory using algae also revealed low concentrations of dissolved silver and cobalt (0.2–0.5 mg/L, corresponding to 0.1–0.5% of 20–100 mg/L Ag NPs) and Co (0.06–0.44 mg/L, corresponding to <0.02% of Co NPs), suggesting that dissolved metals had a low contribution to the toxicity of NPs in algae [51,52]. The control root length of *R. sativus* (115–152 mm) was approximately twice that of the *L. sativa* control (61–74 mm), providing a large surface area for contact with particles or soluble metals. Therefore, among the various causes of the inhibitory effects of NPs on *L. sativa* and *R. sativus* root growth, the available surface area of roots may be strongly related to the contact surface area of NPs, which may be an important factor in NP toxicity. Bakand and Hayes [53] reported the interactions of NPs with biological milieu and found that toxic effects were significantly associated with a large surface area-to-mass ratio and surface characteristics.

Studies examining single NPs may not provide an accurate toxicity assessment because mixtures of contaminants are generally introduced into the environment. Mixtures may have additive, antagonistic, or synergistic effects [54]. Different effects on the root growth of the two plant species were observed depending on the exact binary mixture and NP concentration. The observed root growth inhibition by the high concentrations of binary mixtures was 3–10 times higher (*L. sativa*: 24%, *R. sativus*: 42%) than that for the low concentration groups (*L. sativa*: 8%, *R. sativus*: 4%) (Supplementary Table S1). We determined the interaction effects of the mixtures based on the P(O)-to-P(E) ratios for *L. sativa* (average: 0.62; *p* = 0.0591–0.9250) and *R. sativus* (average: 0.61; *p* = 0.2948–0.9786). The lack of significance (*p* > 0.05) suggested that the NP mixtures had additive effects on the root growth of both plant species. A positive correlation between P(O) and P(E) was observed for *L. sativa* (R^2^ = 0.8615), and less so for *R. sativus* (R^2^ = 0.5237), at a wide range of NP concentrations. The lower correlation for *R. sativus* was not clear; however, the partial solubility of the NPs or longer root length of *R. sativus* may explain this result. Previous studies have observed different modes of toxicity among different test organisms. Both additive (50%) and synergistic (47%) effects on seed germination have been observed, whereas mainly synergistic (67%) and additive (67%) effects have been observed in bacterial bioluminescence tests and algal growth tests, respectively [55,56]. Azevedo et al. [57] suggested that the toxicity of a binary mixture of ZnO and Ag NPs could be predicted based on not only the toxicity of their components but also the interaction between the exact NPs and concentrations used. Future toxicity studies should use several test methods and a wider range of mixtures to fully assess NP toxicity, and should also consider several different physicochemical characteristics of NPs.

**Figure 5:**
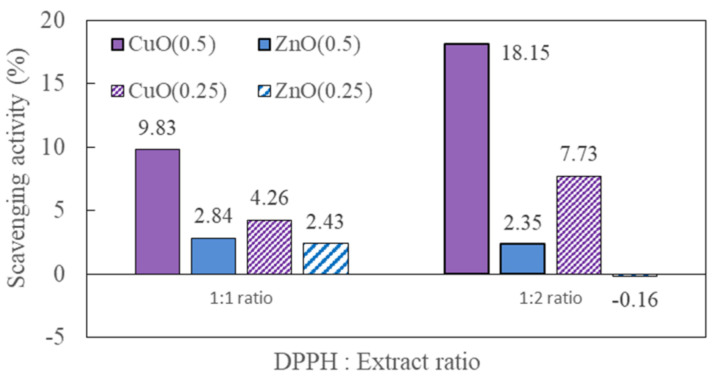
Comparison of DPPH scavenging activity in *Lactuca sativa* at the exposure of different concentration of CuO and ZnO. CuO(0.5) represents CuO exposure at final concentration 0.5 mg/L.

Different mechanisms may be responsible for NP toxicity depending on the tested species and incubation conditions [58]. Although the mechanisms responsible are largely unknown, there may be differences in bioavailability and contact ability among different organisms that influence the effects of NPs [34,59]. For example, a slight impact was observed for Cu ions in solution because the toxicity to plants was due to the accumulation of NPs within cells, and small particles were more reactive because of their high specific surface area and ability to penetrate organisms [60]. Studies have demonstrated that the main mechanism of phytotoxicity is cellular oxidative stress caused by the production of excess ROS, which affects proteins, lipids, carbohydrates, and DNA in plants and alters cell structure and cell membrane permeability [29,30,61,62]. Supporting this, an increase in antioxidant enzyme levels in the presence of NPs has been reported [63,64]. For example, superoxide dismutase activities at 10 and 50 mg/kg CeO_2_ NPs significantly increased 100% more than control treatment in radish leaf [62]. In addition to excess ROS, high NPs concentration can also cause decreases of various phytohormones—such as givverellins, brassinosteroid, and zeatin riboside—that are of importance in plant growth and development [65]. As a preliminary assay for future investigation, antioxidant activity was measured based on the DPPH (diphenyl picryl hydrazyl) radical scavenging activity [23]. Overall results indicated that the DPPH radical scavenging activity is correlated with the plant growth inhibition. The antioxidant activity of high toxic CuO NP in plant growth was approximately 2–8 times higher depending on NP concentration and ratio of methanol extract and DPPH compared to low toxic ZnO NP (Figure 5; Supplementary Table S3). However, more detailed investigation needs to be done in subsequent research. Research also has shown that the toxic effects of partially soluble NPs could be caused by both the solubilized ions and the particles, and may be affected by several factors, such as the concentration, incubation conditions, and type and size of NPs [11,28]. Therefore, an understanding of the interactions of NPs with the test organisms is important. Most studies have used laboratory experiments under controlled conditions that may differ substantially from field conditions; therefore, the establishment of properly designed experiments under environmentally realistic conditions is required to accurately evaluate the environmental impacts of NPs [5].

## 5. Conclusions

In this study, we compared the effects of exposure to NPs, alone and in binary mixtures, on root and shoot growth in two plant species under shaking and non-shaking incubation conditions. The effects of NPs on root and shoot growth differed according to the incubation conditions and plant species. Greater root growth inhibition was observed under shaking than non-shaking conditions, and for *L. sativa* than *R. sativus*. Exposure to binary NP mixtures showed an additive effect on root growth inhibition in both plant species. NP toxicity generally displays large variability in controlled laboratory tests due to their reaction with environmental constituents. Thus, additional interdisciplinary studies by plant, environmental, agricultural, material, and analytical scientists under various modified field conditions and using combinations of sensitive bioassays are needed to assess the long-term and real-time effects of exposure to NPs in soil and water systems.

## Figures and Tables

**Table 1:** Inhibitory effects nanoparticle (NP) exposure on the root and shoot growth of *Lactuca sativa* under non-shaking and shaking incubation conditions.

Incubation Condition		EC_50_ (mg/L)		
		CuO NPs	ZnO NPs	NiO NPs
Non-shaking	Shoot	>5 ^a^ (90%) ^b^	>10 (107%)	>20 (104%)
	Root	1.28 (0.75–2.17) ^c^	1.31 (0.89–1.92)	5.57 (3.41–8.73)
Shaking	Shoot	>1 (28%)	>2 (26%)	>5 (21%)
	Root	0.25 (0.18–0.37)	0.50 (0.36–0.70)	0.57 (0.32–1.02)

^a^ maximum exposure concentration; ^b^ activity at the maximum exposure concentration; ^c^ 95% confidence interval.

**Table 2:** Relative root and shoot growth of *Lactuca sativa* under non-shaking and shaking incubation conditions after exposure to 1000 mg/L TiO_2_ and Al_2_O_3_ nanoparticles (NPs).

Incubation Condition		Relative Growth Activity (%)	
		TiO_2_ NPs	Al_2_O_3_ NPs
Non-shaking	Shoot	106 ± 11.7	108 ± 22.0
	Root	65 ± 16.4	56 ± 10.1
Shaking	Shoot	84 ± 13.0	89 ± 12.3
	Root	99 ± 18.8	86 ± 26.3

## Data Availability

Data can be available upon request from the authors.

## Supplementary Materials

The following are available online at https://www.mdpi.com/article/10.3390/nano11071653/s1, Four pieces of supplementary information are provided. Table S1: The two concentrations (high and low) of three nanoparticles (NPs) used in 12 combinations to study the effect of binary NP mixtures in *Lactuca sativa* and *Raphanus sativus*. Table S2: Soluble metal concentrations from nanoparticles (NPs) under experimental conditions. Table S3: Preliminary results of DPPH scavenging activity (A_517_). Figure S1. Correlations between the root and shoot growth of *Lactuca sativa* under shaking and non-shaking conditions.
