# Supplementary material for: Comparisons of the Effect of Different Metal Oxide Nanoparticles on the Root and Shoot Growth under Shaking and Non-Shaking Incubation, Different Plants, and Binary Mixture Conditions
Source: Nanomaterials (Basel). 2021 Jun 23;11(7):1653. doi: 10.3390/nano11071653 (PMC8305468; doi:10.3390/nano11071653)
Supplement: Supplementary file 1 [file nanomaterials-11-01653-s001.zip › nanomaterials-1252061-supplementary.pdf]

nanomaterials

MDPI

Supplementary Materials

Comparisons of the Effect of Different Metal Oxide Nanoparticles on the Root and Shoot Growth under Shaking and Non-Shaking Incubation, Different Plants and Binary Mixture Conditions

In Chul Kong [1], Kyung-Seok Ko [2,*] and Dong-Chan Koh [2]

[1] Department of Environmental Engineering, Yeungnam University, Gyungsan 38541, Korea; ickong@ynu.ac.kr
[2] Geologic Environment Division, Korea Institute of Geoscience & Mineral Resources (KIGAM), Daejeon 34132, Korea; chankoh@kigam.re.kr
* Correspondence: kyungsok@kigam.re.kr; Tel.: +82-42-868-3162; Fax: +82-42-868-3414

Table S1. The two concentrations (high and low) of three nanoparticles (NPs) used in 12 combinations to study the effect of binary NP mixtures in *Lactuca sativa* and *Raphanus sativus*.

Plant	NPs (mg/L)			
	CuO	ZnO	NiO	Combinations
L. sativa	0.12[a], 0.06[b]	0.25, 0,12	0.30, 0.15	12
R. sativus	0.18, 0.09	0.62, 0.31	1.42, 0.71	12

[a] high test concentration; [b] low test concentration.

Table S2. Soluble metal concentrations from nanoparticles (NPs) under experimental conditions.

Conditions	CuO		ZnO		NiO	
	NPs (mg/L)	Soluble metal (μg/L)	NPs (mg/L)	Soluble metal (μg/L)	NPs (mg/L)	Soluble metal (μg/L)
Lactuca sativa	0.2	10 ± 1 (5%)	0.2	12 ± 3 (6%)	0.5	4 ± 1 (0.8%)
	0.5	19 ± 6 (3.8%)	0.5	33 ± 9 (6.6%)	1.0	10 ± 1 (1%)
	1.0	22 ± 7 (2.2%)	1.0	65 ± 36 (6.5%)	2.0	34 ± 5 (1.7%)
	2.0	57 ± 20 (2.9%)	2.0	120 ± 59 (6.0%)	5.0	156 ± 16 (3.1%)
Raphanus sativus	0.1	4 ± 2 (4%)	0.5	9 ± 2 (1.8%)	1.0	3±2 (0.3%)
	0.2	11 ± 8 (5.5%)	1.0	23 ± 13 (2.3%)	2.0	4±1 (0.2%)
	0.5	21 ± 3 (4.2%)	1.5	42 ± 31 (2.8%)	4.0	17±6 (0.4%)
	1.0	40 ± 1 (4%)	2.0	55 ± 36 (2.75%)	5.0	37±20 (0.7%)

Table S3. Preliminary results of DPPH scavenging activity (A_{517}).

Absorbance (517 nm)	Control	CuO (mg/L)		ZnO (mg/L)	
		0.5	0.25	0.5	0.25
Extract (no DPPH)	0.025	0.018	0.019	0.022	0.024
DPPH:extract (1:1)	1.012	0.908	0.964	0.981	0.987
DPPH:extract (1:2)	0.620	0.505	0.568	0.603	0.621

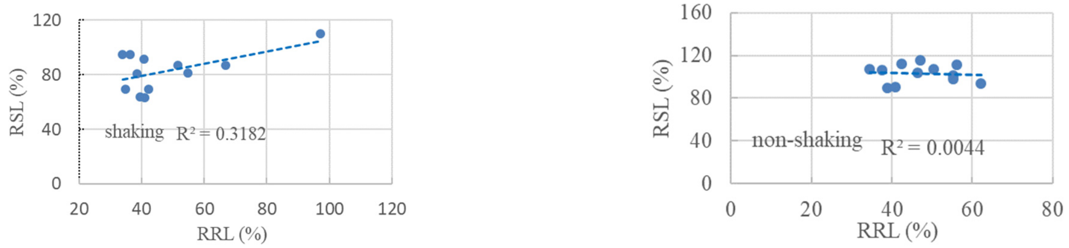

Figure S1. Correlations between the root and shoot growth of *Lactuca sativa* under shaking and non-shaking conditions.
